# 

**Published:** 1802-02

**Authors:** 


### To CORRESPONDENTS.

*We are sorry that Mr. Ward's request to have his paper returned for alteration did not come into the Editors hands till after it was printed off. As Mr. Ward appears desirous of avoiding unnecessary controversy, we entreat our readers to suppose the paper not published, till Mr. W. has made such additions and alterations as he may judge proper.*

*Mr. Mann's letter shall be sent as desired.*

*Mr. Cam's Case of Monstrosity has not been received.*

*The Indices are not made by the Editors, but they will be happy to correct any error pointed out to them. They could never doubt Mr. Hope's veracity or accuracy; and if the Index, or running Titles (which we again request our Correspondents to add to their own papers) convey Ideas different from those* intended *by the Writers, it is not by design.*

*R. M's difficulties in the Brunonian System are too metaphysical for our Journal.*

*G. W's request is inadmissible. To attempt to comply with it would be Quackery.*

*An experienced practitioner, after due inquiries in person, can alone answer him.*

*Communications are received from Drs. Cogan, Brown, and White; Messs. Howard, Cooke, Crowthers, and Philosophus, for whose name Dr. B. will be obliged.*

